# Supplementary material for: Effect of Low-Frequency Renal Nerve Stimulation on Renal Glucose Release during Normoglycemia and a Hypoglycemic Clamp in Pigs
Source: Int J Mol Sci. 2024 Feb 7;25(4):2041. doi: 10.3390/ijms25042041 (PMC10888375; doi:10.3390/ijms25042041)
Supplement: Supplementary file 1 [file ijms-25-02041-s001.zip › ijms-2800186-supplementary.pdf]

Number of animal	1		2		3		4		5		6		7	
Body side of stimulation	left	right	left	right	left	right	left	right	left	right	left	right	left	right
	nd	nd	0,37	-0,50	0,11	-0,13	0,20	0,59	0.24	0.27	0.07	-0.27	0.59	0.14
	0.39	0.44	2.01	-0.17	0.16	0.36	0.39	0.35	0.26	0.2	0.33	0.02	0.31	-0.08
	0.30	-0.04	0.28	0.13	0.04	0.23	0.01	-0.19	0.09	-0.52	0.29	-0.5	-0.3	0.14
	0.31	0.34	0.07	0.08	0.1	0.15	0.15	0.47	0.3	-0.19	0.16	-0.39	0.0	0.02
	0.19	-0.28	0.18	-0.17	0.05	0.09	0.74	0.53	-0.38	-0.3	0.0	0.53	0.64	-0.36
	0.74	0.19	0.25	0.26	0.17	0.08	0.09	0.18	0.05	0.05	0.31	0.41	0.13	0.2
Hypoglycemia	0.26	0.52	0.24	0.31	0.12	0.12	0.18	0.11	0.08	0.17	0.68	0.46	0.18	0.18
	0.39	0.37	0.24	0.21	0.09	0.04	0.15	0.1	0.16	0.18	0.87	0.63	0.11	0.24
	0.28	0.34	0.25	0.26	0.09	0.05	0.38	0.46	0.3	0.29	0.72	0.43	0.22	0.27
	0.31	0.32	0.36	0.34	0.08	0.08	0.14	0.11	0.3	0.27	0.62	0.29	0.4	0.36
	0.28	0.31	0.16	0.14	0.16	0.13	0.39	0.58	0.26	0.22	0.67	0.23	0.75	0.45

Table S1. Original data of side-dependent renal glucose release in mmol/l, episodes of hypoglycemia are highlighted in light gray and episodes of 15-minute low frequency renal stimulation in dark gray.

SGN

Variable	Value	Std.Error	DF	t-value	p-value
Intercept	0.01320309	0.006063057	74	2.177629	0.0326
Stimul	0.01714802	0.010566148	74	1.622921	0.1089
Hypoglyk	0.02940364	0.008574458	68	3.429212	0.0010
Stimul:Hypoglyk	-0.03012008	0.016828346	74	-1.789842	0.0776

SRP

Variable	Value	Std.Error	DF	t-value	p-value
Intercept	165.04466	26.35264	74	6.262926	0.0000
Stimul	-27.78591	16.38879	74	-1.695421	0.0942
Hypoglyk	15.05001	28.43733	68	0.529234	0.5984
Stimul:Hypoglyk	-35.98923	27.19634	74	-1.323311	0.1898

GFR

Variable	Value	Std.Error	DF	t-value	p-value
Intercept	23.067527	3.809733	74	6.054893	0.0000
Stimul	-6.774361	2.788799	74	-2.429132	0.0176
Hypoglyk	16.757777	3.670796	68	4.565162	0.0000
Stimul:Hypoglyk	-6.123723	4.598385	74	-1.331711	0.1870

Urinary Na

Variable	Value	Std.Error	DF	t-value	p-value
Intercept	106.98868	4.585962	74	23.329602	0.0000
Stimul	-13.12459	1.876240	74	-6.995158	0.0000
Hypoglyk	-7.23375	2.146225	68	-3.370455	0.0012
Stimul:Hypoglyk	2.34161	3.079605	74	0.760361	0.4495

Urine volume

Variable	Value	Std.Error	DF	t-value	p-value
Intercept	33.39348	4.288784	74	7.786234	0.0000

Variable	Value	Std.Error	DF	t-value	p-value
Stimul	-9.84710	2.744522	74	-3.587910	0.0006
Hypoglyk	-10.34753	4.674727	68	-2.213504	0.0302
Stimul:Hypoglyk	-1.16733	4.552823	74	-0.256397	0.7984

PAH

Variable	Value	Std.Error	DF	t-value	p-value
Intercept	980.0520	211.9308	74	4.624395	0.0000
Stimul	325.3348	110.2823	74	2.950019	0.0043
Hypoglyk	140.5117	178.4706	68	0.787310	0.4338
Stimul:Hypoglyk	-55.3655	182.8190	74	-0.302843	0.7629

Inulin

Variable	Value	Std.Error	DF	t-value	p-value
Intercept	416.2649	58.14078	74	7.159602	0.0000
Stimul	52.7942	44.21025	74	1.194163	0.2362
Hypoglyk	248.4808	81.71049	68	3.040991	0.0033
Stimul:Hypoglyk	136.1258	73.39742	74	1.854640	0.0676

Table S2. Comprehensive statistical data on the linear mixed model parameters.
